# Moral Causes of Cholera

**Published:** 1855-01

**Authors:** 


### MORAL CAUSES OF CHOLERA.

The London Christian Times suggests that moral causes have much to do in engendering this disease; and that moral remedies may go far to alleviate or cure it:

The filthy, low-lying regions, says the Times, where the disease presents itself with most inveteracy, are also the regions of coarse, imbruted vice. Self-indulgence in sordid and unwholesome luxuries undermines the constitution. Perseverance in such indulgence for a series of generations debilitates a race.—The harass and anxiety attendant upon precarious and dishonest means of obtaining a livelihood, shake terribly such enfeebled constitutions.

Vicious indulgence and sordid habits by demoralizing a large proportion of the lower classes, are the real cause of the predisposition to a new and awful form of disease.—The filth and squalor are merely the external indications of this internal rottenness. When a large portion of any community has been thus pre-disposed, disease catches around it like wild-fire, and even those who have kept themselves above the general degradation are not exempted from its visitations. The honest poor are by their poverty brought into contagious proximity to the class prepared for sickness. The wealthy are brought into contact with the infected stratum of society by business-relations. Let the whole truth be told: the vicious and the unreflecting of the wealthier class expose themselves to contagion by visiting infected dens in search of illicit pleasure. Nay, more; the anxious, mammon-hunting, voluptuous habits of the wealthy predispose them to contagion.

Moral causes of disease can only be combated by counter-agents. It is not meant that physical remedies and lenitives for cholera are to be dispensed with, but that moral remedies and lenitives are to be superadded.

